# Assessment of cytotoxicity and antioxidant properties of berry leaves as by-products with potential application in cosmetic and pharmaceutical products

**DOI:** 10.1038/s41598-021-82207-2

**Published:** 2021-02-05

**Authors:** Aleksandra Ziemlewska, Martyna Zagórska-Dziok, Zofia Nizioł-Łukaszewska

**Affiliations:** Department of Technology of Cosmetic and Pharmaceutical Products, Medical College, University of Information Technology and Management in Rzeszow, Kielnarowa 386a, 36-020 Tyczyn, Poland

**Keywords:** Cell biology, Plant sciences

## Abstract

*Vaccinum myrtillus L., Ribes nigrum L., Rubus fruticosus L., Fragaria vesca L.* leaves are considered an agro-waste of the berry industry. Although numerous studies indicate fruit is a rich source of bioactive compounds, the authors prove leaves can also be a valuable source of compounds used in the pharmaceutical and cosmetic industries. The study attempts to assess and compare the antioxidant and cytotoxic properties of berry leaves extracts. The total phenolic compounds, flavonoids, anthocyanins and procyanidins content were determined. Antioxidant potential was evaluated using the DPPH radical scavenging method. Cytotoxicity studies were conducted to evaluate the effect of the extracts on the metabolism and proliferation of keratinocytes and fibroblasts in vitro. The effect on the migration capacity of these cells was also assessed. The obtained results show that the examined extracts are a source of valuable bioactive agents. All tested extracts show significant ability to remove free radicals in higher concentrations. Cytotoxicity assessments have shown that leaf extracts of the analyzed plants differ in cytotoxicity, both for keratinocytes and fibroblasts. The results of the assessment of cell migration capacity correlate with cytotoxicity tests, because the concentration of extracts showing cytotoxic activity towards the tested cells also inhibited their migration.

## Introduction

The skin is constantly exposed to adverse external factors that contribute to the increased formation of free radicals. The right line of defense is using substances that show strong antioxidant activity. Their activity is most often determined by the presence of polyphenolic compounds^1–4^, which have a positive effect on a number of enzyme systems and functional proteins, cell signaling or maintaining or restoring the redox balance, which can be disturbed as a result of oxidative stress^5,6^. Due to the multidirectional impact of antioxidants on the human body, the pharmaceutical and cosmetics industry has been looking for substances exhibiting strong antioxidant activity for many years. These substances may include raw materials of plant origin, which in addition to antioxidant activity, contain a number of active substances that have a soothing, regenerating or anti-inflammatory effect and can play the role of auxiliary substances contributing to the stability or bioavailability of cosmetic preparations^7–10^.

Antioxidants of natural origin can be found in many plants in their different parts. A particular attention should be paid to those parts of the plant used as a plant raw material, which are not often perceived as pro-health raw materials having functional properties^11^. It is particularly important from the industry perspective, as the content of plant material as a substance of potential industrial significance is important not only because of its multifunctionality and exhibiting a positive effect on the body, but also because of the economic factors. Therefore, it is important that rational management should span not only over raw materials and products but also over by-products and waste generated at various stages of the production process^12–14^. Currently, the demand for this type of multifunctional plant-based raw materials with a broad spectrum of activity is increasing. While the fruits of such plants as *Ribes nigrum L., Vaccinium myrtillus L., Rubus fruticosus L., Fragaria vesca L.* are valued and willingly used, the leaves of these plants are not widely used, interest in their use is on an initial stage at the moment. Due to the high content of various phenolic compounds, extracts from the leaves of these plants and essential oils have strong antioxidant properties. Studies conducted with currant leaf extract have shown that it has stronger antioxidant properties compared to the extract of buds or currant fruit. It is characterized by the presence of compounds such as quercetin, myricetin, rutin and phenolic acids such as salicylic, p-coumaric, gallic, chlorogenic or ferulic acid^15–17^.

In contrast, blueberry leaves contain a significant amount of phenolic glycosides such as arbutin, anthocyanins, catechins, flavonols and hydroxycinnamic acid. Their content increases when the leaves are exposed to direct sunlight. It has also been shown that anthocyanins present in the leaves significantly affect the mitigation of the negative impact of solar radiation on human fibroblasts, which may contribute to reducing the photoaging process of the skin and inhibiting collagen degradation^18,19^.

Leaves of blackberry and wild strawberries analyzed in this paper are also characterized by a high content of bioactive compounds. They contain phenolic compounds such as chlorogenic, ellagic, coffee, epicatechin, quercetin, kaempferol but also proanthocyanidins, triterpenes. Blackberry leaf extract has been shown to slow down skin aging due to inhibition of metalloproteinases that catalyze the degradation of intercellular matrix proteins^5,20–22^.

At present, from the “circular economy” perspective, there is an increased interest to use agricultural waste resources to produce high-value compounds. *Vaccinum myrtillus L., Ribes nigrum L., Rubus fruticosus L., Fragaria vesca L.* leaves are considered essentially an agro-waste of the berry industry. Many research shows that the fruits of these plants are a rich source of bioactive compounds and have beneficial health properties, therefore from an economic and ecological point of view, it is worth looking at by-products as compounds that might be widely used in the pharmacy and cosmetics industry.

During the research, the biochemical and cytotoxic properties of the obtained extracts were assessed. The antioxidative potential of the tested products and the content of bioactive compounds such as phenolic compounds, flavonoids, anthocyanins and procyanidins were determined. In addition, the cytotoxic effect of the obtained extracts on skin cells (keratinocytes and fibroblasts) and the regenerative effect (scratch test using cell cultures) was determined.

## Materials and methods

### Plant material and extraction procedure

Plant material was purchased from Dary Natury—a polish manufacturer and distributor of herbs. Leaves *of R. nigrum L., V. myrtillus L., R. fruticosus L., F. vesca L.* were collected on controlled and ecological plantations. No chemical fertilizers nor plant protection products were used for the cultivation. In addition, after obtaining the plant material, a preliminary selection of plant material was carried out, paying particular attention to chemotaxonomic factors. Plant leaves were collected in August 2019. Then extracts were made separately for analyzed plants. Each sample was extracted using an 80:20 mixture of water and glycerin as a solvent. Extracts from plants of those plants were obtained using an ultrasound-assisted extraction (UAE). UAE was performed according to the method described by Yang et al. in ultrasonic bath (Digital Ultrasonic Cleaner) equipped with time controller^23^. The mixtures were homogenized at room temperature for 50 min (5 cycles for 10 min). The obtained extracts were then collected and filtered three times through Whatman No. 10 filter paper using a vacuum pump. The extracts were stored in the dark at 4 °C until further analysis.

### Total phenolic content determination

The concentration of total phenolic compounds in extracts were determined spectrophotometrically using the Folin-Ciocalteu method described by Singleton et al. with some modification using gallic acid (GA) as a standard^24^. This method depends on the reduction of Folin’s reagent by phenols to a mixture of blue oxides with a maximum absorption of about λ = 740 nm. Briefly, 300 μL tested extract samples and standard solution of varying concentrations were mixed with 1500 μL of 1:10 Folin Ciocalteu reagent and thoroughly mixed. After 6 min of incubation, 1200 μL of 7.5% (w/v) Na_2_CO_3_ solution were added to each sample then promptly mixed and incubated in the dark at room temperature for 2 h. The deionized water was used for dilution and control sample. The absorbance was read at λ = 740 nm on AquamateHelion (Thermo Scientific) spectrophotometer. The total phenolic concentration in analyzed extracts were calculated from a gallic acid (GA) calibration curve (10–100 mg/mL). The estimation of phenolic compounds in the extracts was carried out in triplicate and the results were averaged.

### Total flavonoids content determination

The concentration of flavonoid content in extracts were analyzed spectrophotometrically using aluminium nitrate nonahydrate by method described by Matejić et al. with modifications^25^. Samples for determination was prepared by mixing the different concentrations of extracts and mixture consisting of 80% (v/v) C_2_H_5_OH, 10% Al(NO_3_)_3_ × 9 H_2_O (w/v) and 1 M C_2_H_3_KO_2_ (w/v). 2400 μL the previously prepared reaction mixture was mixed with 600 μL of tested extract sample and standard solution in various concentrations. The deionized water was used for dilution and control sample. After 40 min of incubation at 25 °C, the absorbance was read at λ = 415 nm on FilterMax F5 AquamateHelion (Thermo Scientific) spectrophotometer. The total flavonoid concentration in examined extracts were calculated from a quercetin hydrate (Qu) calibration curve (10–100 mg/L). Measurements were made in triplicate.

### Determination of total procyanidin content

The total content of procyanidin in the leaf extracts of *Vaccinum myrtillus L., Ribes nigrum L., Rubus fruticosus L.* and *Fragaria vesca L.* was obtained using the DMAC method proposed by Payne et al. with some modification^26^. To prepare the DMAC solution, HCl was initially mixed with alcohol in a ratio of 3:27 (v/v). After cooling the solution prepared in this way at 4 °C, 4-(dimethylamino) cinnamaldehyde (DMAC) was added, reaching a final concentration of 0.1% (w/v), and mixed thoroughly. Procyanidin B2 dissolved in a methanol-aqueous solution was used to prepare the standard curve (1–1000 ppm). The extracts were extracted in a mixture of acetone–water–acetic acid (70 + 29.5 + 0.5, v/v/v) on a shaker and then sonicated at 37 °C for 30 min. Samples were then vortexed twice with 30-min breaks between consecutive mixes. In the next stage, the tubes were centrifuged for 5 min at 3000 rpm, and then filtered through membrane filters. Dilutions of the analyzed berry leaf extracts were prepared in alcohol. Alcohol was used as the blank. All samples (blank, standard solutions and individual dilutions of extracts) were mixed with the previously prepared DMAC solution in the ratio 5:25 (v/v). The absorbance of the samples was then immediately measured spectrophotometrically at λ = 640 nm every 5 min for 30 min.

### Determination of total anthocyanin content (TAC)

The anthocyanin content in the analyzed berry leaf extracts was tested using the pH-differential method described by Le et al. and Sutharut and Sudarat^27,28^. Initially, the test extracts were mixed with 2 N HCl and ammonia in a ratio of 1: 1 (v/v). The procedure is based on the structural alteration of the anthocyanin chemical forms, which is assessed by measuring absorbance at pH 1.0 and 4.5. These pigments undergo reversible structural changes and the change in pH manifests itself in strikingly different absorbance spectra. The berry extracts tested in this study were diluted with 0.025 M hydrochloric acid—potassium chloride buffer (pH = 1) and 0.4 M sodium acetate buffer (pH = 4.5). Each of the analyzed samples (all 4 extracts separately) was diluted with buffers to obtain an absorbance value in the range of 0.2–1.4. The absorbance of all tested samples was measured at λ = 510 nm and λ = 700 nm on an AquamateHelion (Thermo Scientific) spectrophotometer. Distilled water was used as a blank sample. Measurements were made every 5 min for a period of 1 h. The absorbance of the diluted sample (A) was calculated using the equation:

$$A=(A510-A700) pH 1.0 - (A510-A700)pH 4.5$$

The concentration of the monomeric anthocyanin pigment in analyzed extracts were calculated using the following equation:

$$Monomeric \, anthocyanin \, pigment \, \left( {mg/L} \right) \, = \, = \, \left( {A \, \times \, MW \times \, DF \, \times \, 1000} \right)/\left( {\varepsilon \times 1} \right)$$

where: MW is the molecular weight , DF is the dilution factor, and ε is the molar absorptivity.

The pigment content was calculated as cyanidin-3-glucoside, whose MW = 449.2 and ε = 26.900. Then, the content was calculated and expressed in mg of total anthocyanin content / 100 g of sample.

### DPPH radical scavenging assay

The free radical activity of analyzed plant extracts were determined by using the stable 1,1-diphenyl-2-picrylhydrazyl (DPPH). This method was adopted by Brand-Williams et al.^29^. Volume 33 μl of tested plant samples at various concentrations (0.5–10.0%) were mixed with 167 μl methanolic solution of DPPH (4 mM) in a 96-well plate. DPPH solution (0.1 mL) plus ethanol 96% v/v (EtOH) (3.9 mL) was used as a negative control. The analyzed samples were shaken vigorously and absorbance of the remaining DPPH radical was measured at λ = 517 nm in every 5 min for 30 min on UV–ViS Filter Max 5 (Thermo Scientific) spectrophotometer. Measurements were carried out in triplicate for each sample. DPPH solution mixed with equal volume of distilled water was used as a control. The antiradical activity of the plant extracts were expressed as inhibition percentage using the following equation:

$$\% DPPH \cdot scavenging=\frac{Abs control-Abs sample}{Abs control}\times 100\%$$

where Abs control is the absorbance of the control sample (containing all reagents except the test extract or standard), and Abs sample is the absorbance of the test extract or standard.

### Cell culture

BJ cells (fibroblasts, ATCC CRL-2522) used in the study were obtained from the American Type Culture Collection (Manassas, VA 20,108, USA). HaCaT cells (normal human keratinocytes) were purchased from CLS Cell Lines Service (Germany). Both cell lines were maintained in a DMEM (Dulbecco’s modified essential medium, Gibco) with L-glutamine, supplemented with 10% (vol/vol) FBS (fetal bovine serum, Gibco), and 1% (vol/vol) antibiotic (100 U/ml Penicillin and 1000 µg/ml Streptomycin, Gibco). The cultured cells were kept at 37 °C in a humidified atmosphere of 95% air and 5% of carbon dioxide (CO_2_). When the cells reached confluence, the culture medium was removed from the flask (Corning) and cells were rinsed two times with sterile PBS (Phosphate-Buffered Saline, Gibco). The confluent layer of cells was trypsinized using Trypsin/EDTA (Gibco) and then resuspended in fresh medium (DMEM or MEM, depending on the cell type). Cells prepared in this way were treated with different concentrations of the analyzed berry leaf extracts^30^.

### Cell viability assay

#### Neutral red uptake assay

The neutral red uptake test (Sigma Aldrich) was used in studies to assess the viability of skin cells, HaCaT and BJ, treated with the tested extracts. This test is based on the protocol described by Borenfreund et al.^31^. This test allows assessing the cytotoxicity of extracts by evaluating the ability of the cells tested to bind a neutral red dye in lysosomes. Cells in fresh DMEM medium were plated into 96 well plates with a density of 1 × 10^4^ cells for each well. After 24 h of incubation, the medium was removed and tested concentrations (0.5, 1.0, 2.5, 5.0 and 10.0%) of leaf extracts of four different fruits (*Vaccinum myrtillus L., Ribes nigrum L., Rubus fruticosus L.* and *Fragaria vesca L*) were added to each well. The treated cells were grown in an incubator for another 24 h. The control group were cells treated with DMEM medium without the addition of extracts. After 24 h of exposure of the cells to the extracts, HaCaT and fibroblast cells were incubated for 2 h with a neutral red dye (40 μg/ml) which was dissolved in serum-free medium (DMEM). In the next step, the cells were washed twice with phosphate buffered saline (PBS) and then 150 μl decolorizing buffer (EtOH/AcCOOH/H_2_O, 50%(v/v)/1%(v/v)/49%(v/v)) was added to each well. To extract neutral red from cells, both fibroblasts and keratinocytes were shaken for 15 min. Neutral red dye uptake was determined by measuring the optical density (OD) at λ = 540 nm in a FilterMax F5 microtiter plate spectrophotometer (Thermo Fisher). The experiments were performed in four replicates for each tested extract concentration and presented as a percentage of the control value (100%).

#### Alamar blue assay

Another test used to assess the cytotoxicity of the tested extracts and check their effect on cell viability was the Alamar Blue assay (Sigma, R7017). This assay is based on the initial protocol described by Page et al.^32^. In the first step, keratinocytes and fibroblasts were seeded at a density of 10^4^ cells per well in transparent 96-well plates and cultured in DMEM medium until appropriate confluency was achieved. Then, cells were exposed to various concentrations of leaf extracts (0.5% to 10.0%) and incubated for 24 h. The control group were cells cultured in DMEM medium without the addition of extracts. After 24 h exposure, the extracts diluted in medium were removed and a resazurin solution (250 μL/ well) with a final concentration of 60 μM resazurin was added to the wells and incubated for 2 h at 37 °C in the dark. Absorbance at λ = 570 nm was then measured using a microplate reader (FilterMax F5, Thermo Fisher). Cytotoxicity was assessed in three independent experiments in which each extract concentration was analyzed in four replicates. The results were expressed as the percentage of viability of the control cells whose viability was defined as 100%.

### Scratch wound assay

The next stage of the study was to assess the possibilities of water-glycerin extracts obtained from the leaves of the four plants studied to stimulate keratinocytes and fibroblasts migration. For this purpose, a widely used scratch test was carried out. Initially, the cell lines analyzed were cultured in 6-well plates in DMEM medium with L-glutamine, glucose, sodium pyruvate and the addition of 10% FBS and antibiotics. Cells were cultured in an incubator at 37 °C. After the keratinocytes and fibroblasts reached the cell monolayer with appropriate confluence, the cell layer was carefully scratched with a 10 μl pipette tip to make a break in the monolayer. Then, the cells were treated with water-glycerin extracts from the leaves of *Ribes nigrum L, Vaccinium myrtillus L, Rubus fruticosus and Fragaria vesca L.* in the concentration range of 0.5–10.0%. Test extracts were diluted in DMEM medium that was used during cell culture, but the FBS content was reduced to 1%. After 24 h of incubation, photos of cultured fibroblasts and keratinocytes treated with the tested leaf extracts were taken. For this purpose 10 × microscope magnification was used.

### Statistical analysis

Each value is the mean of three replicates. Obtained values were presented as mean ± SD. Significant differences between obtained values were analyzed using GraphPad Prism 5.0 software using One-way ANOVA and Tukey’s test. The differences were considered significant when p < 0.05.

## Results and discussion

Reactively oxidized molecules are continuously produced by cellular processes. These include ROS (reactive oxygen species) and RNS (reactive nitrogen species). ROS include free radicals such as hydroxyl (OH), superoxide (O2^–^) and non-free radicals such as hydrogen peroxide (H_2_O_2_). RNS include free radicals such as nitric oxide, nitrogen dioxide as well as peroxynitrite (OONO^–^). Any disturbance of the balance of antioxidants and pro-oxidants resulting from the increased production of reactive forms leads to oxidative stress in our body^33,34^. In order to prevent any oxidative changes, plant antioxidants in the form of polyphenols are used, which normalize the concentration of enzymes involved in carbohydrate metabolism. Plant polyphenols are classified as secondary plant metabolites. Many studies indicate their beneficial health properties. They contain the hydroxyl group, and by donating hydrogen and forming stable radical intermediates, they play an important role in the antioxidant effect^35,36^.

### Assessment of the total content of different groups of plant compounds

The most popular methods for evaluation of the antioxidant activity and phenolic compounds of extracts from *R. nigrum L., V. myrtillus L., R. fruticosus L., F. vesca L.* are the Folin–Ciocalteu method and DPPH (2,2-diphenyl-1-picrylhydrazyl) radical scavenging method. The Folin-Ciocalteu method is a frequently used analytical method due to the high repeatability and accuracy of the results obtained. The total polyphenol content was determined using catechin and gallic acid as a quantitative standard^37,38^. In our research, the total phenolic content in the extracts from the leaves of *R. nigrum L., V. myrtillus L., R. fruticosus L., F. vesca L*. were calculated using the linear regression equation of the gallic acid standard curve (y = 0.0111x + 0.0469, R^2^ = 0.9935). The total content of flavonoids in the analyzed plant extracts determined by spectrophotometry using anhydrous aluminum nitrate and calculated from the linear regression equation of the standard quercetin curve (y = 0.0135x + 0.0009, R^2^ = 0.9980). Both the highest content of phenolic compounds and flavonoids were recorded in the *F. vesca L*. extract (148.48 ± 3.12 mg/g DW and 18.19 ± 0.27 mg/g DW, respectively), while the lowest level of polyphenols was found in the *R. nigrum L*. extract (70.57 ± 2.24 mg/g DW). In this study, the total phenolic compounds of the various extracts from analyzed plants were found to be in the ranges of 70.57 ± 2.24–148.48 ± 3.12 mg/g (as gallic acid equivalent). Analyzes aimed at assessing the total content of proanthocyanidins using the DMAC method, in which we used procyanidin B2 as a standard, showed that the most rich source of these compounds among the tested are *Fragaria vesca L.* leaves, in which the content was 19.03 ± 1.77 mg/g DW. The tested extracts also turned out to be a rich source of anthocyanins, the content of which was assessed using the pH-differential method. This content varied depending on the tested extract. The greatest amount of anthocyanins was detected in extracts of *Rubus fruticosus L*. and *Fragaria vesca L.* − 44.83 ± 2.74 and 49.13 ± 1.99 mg/g DW, respectively (Table 1). These phenolic compounds may directly contribute to the antioxidative activity^39^.

**Table 1:** Total content of phenolic compounds, flavonoids, anthocyanins and procyanidins in leaf extracts of *R. nigrum L., V. myrtillus L., R. fruticosus L.* and *F. vesca L*. expressed as mg/g DW (dry weight). Values are mean of three replicate determinations (n = 3) ± SD.

**Total phenolic compounds content (mg/g DW ± S.D.)**			
*Ribes nigrum L.*	*Vaccinium myrtillus L.*	*Rubus fruticosus L.*	*Fragaria vesca L.*
70.57 ± 2.24	76.73 ± 2.38	121.87 ± 2.11	148.48 ± 3.12
**Total flavonoids content (mg/g DW ± S.D.)**			
*Ribes nigrum L.*	*Vaccinium myrtillus L.*	*Rubus fruticosus L.*	*Fragaria vesca L.*
7.93 ± 0.44	16.36 ± 0.47	17.49 ± 11.74	18.19 ± 0.27
**Total procyanidin content (mg/g DW ± S.D.)**			
*Ribes nigrum L.*	*Vaccinium myrtillus L.*	*Rubus fruticosus L.*	*Fragaria vesca L.*
6.43 ± 0.24	8.03 ± 0.76	13.22 ± 1.06	19.03 ± 1.77
**Total anthocyanin content (mg/g DW ± S.D.)**			
*Ribes nigrum L.*	*Vaccinium myrtillus L.*	*Rubus fruticosus L.*	*Fragaria vesca L.*
28.07 ± 3.08	31.02 ± 0.98	44.83 ± 2.74	49.13 ± 1.99

### DPPH radical scavenging assay

**Figure 1:**
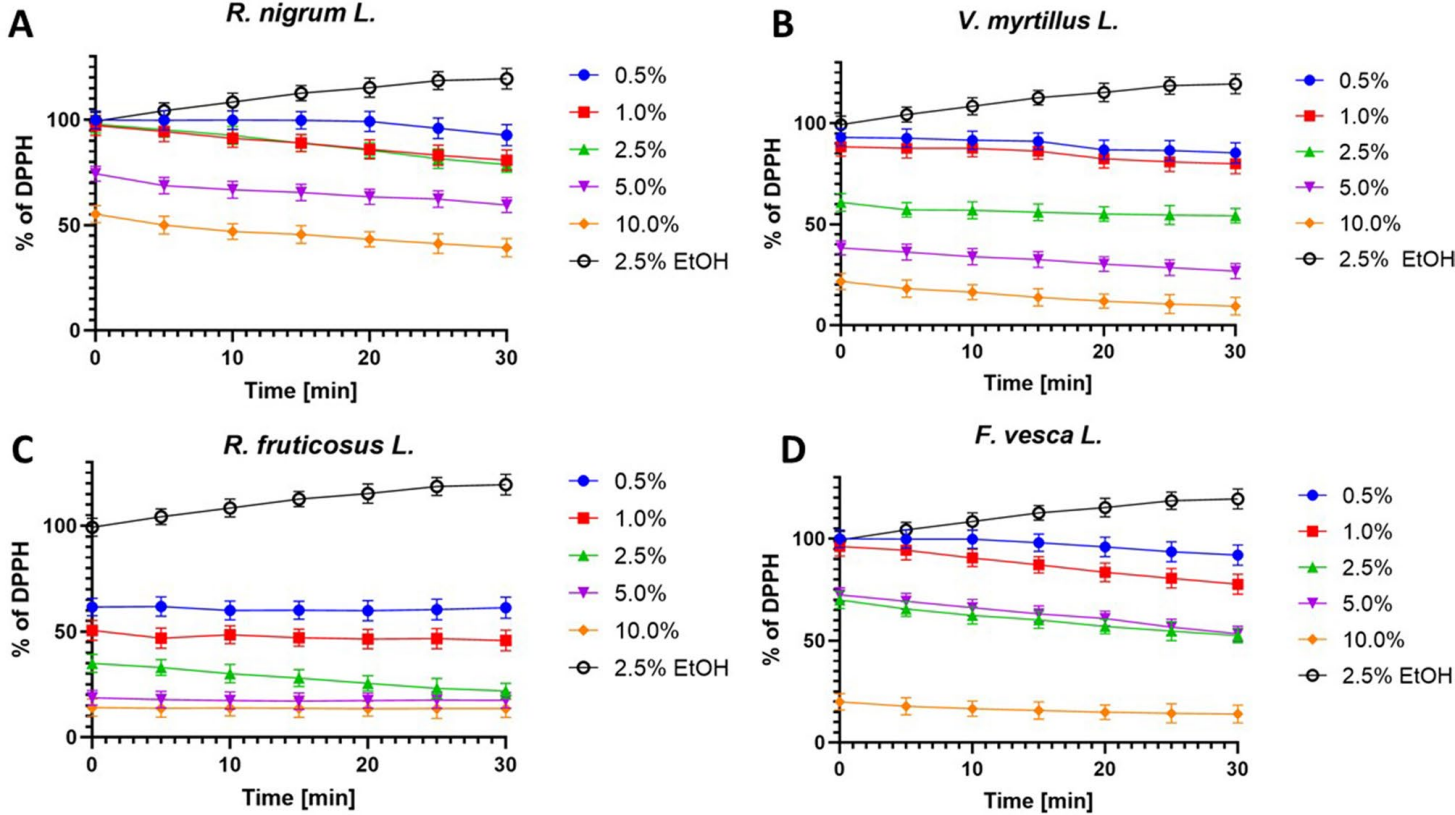
Kinetics of the absorbance changes in DPPH• solutions in the presence of various concentrations (0.5–10.0%) of water-glycerine extracts of *R. nigrum L.* (**A**)*, V. myrtillus L.* (**B**)*, R. fruticosus L.* (**C**)*,* and *F. vesca L*. (**D**). Values are mean of three replicate determinations (n = 3) ± SD. Negative control is: 0.25% ethanol solution.

The DPPH (2,2-diphenyl-1-picrylhydrazyl) scavenging capacity is a measure of the antioxidant capacity of the sample. When DPPH accepts an electron donated by an antioxidant compound, DPPH is reduced and loses its purple color, turning yellow in the presence of oxidants, which can be quantified from changes in absorbance^40,41^. The results of the assay for antioxidant activity are shown in Fig. 1.

In order to determine the antioxidant properties of the extracts tested, five different concentrations were used spanning from 0.5 to 10.0%. Measurements were taken every 5 min for 30 min. The results reveal the percentage inhibition of DPPH radical was found to be increased with concentration. The dependence of scavenging free radicals from time has also been observed. It has been shown that after a certain period of time the percentage of scavenging of free radicals increases. All tested extracts from analyzed plants show significant ability to remove free radicals in higher concentrations. The strongest free radical scavenging activity was observed for the extract of leaves of *R. fruticosus L*. Even at low concentrations, the ability to remove free radicals appears significant. Both for *R. fruticosus L., V. myrtillus L.* and *F. vesca L* after 30 min of the test the level of reduced DPPH• reached almost 90% for highest analyzed concentration (10.0%). The lowest free radical scavenging activity was observed for the extract of leaves of *R. nigrum L.* At the concentration of 10.0% the level of scavenged radicals was about 60%. In addition values of IC_50_ for tested extracts were measured (Table 2). The highest values were observed for water-glycerine extract with *R.nigrum L*., while the lowest values were observed for *R. fruticosus L.* Moreover, for all investigated extracts a decrease of IC_50_ in time was observed.

**Table 2:** Values of IC_50_ for leaf extracts of *R. nigrum L., V. myrtillus L., R. fruticosus L. and F. vesca L*. after different time of exposure.

Extract plant	Time [min]						
	0	5	10	15	20	25	30
	IC_50_						
*R. nigrum* L.	11.36	10.00	9.09	8.91	8.26	7.92	7.50
*V.myrtillus* L.	3.75	3.47	3.23	3.13	2.98	2.87	2.85
*R. fruticosus* L.	1.04	0.87	0.93	0.86	0.85	0.86	0.85
*F. vesca* L.	6.96	6.55	6.37	6.10	5.87	5.53	5.35

Many authors have shown that the extract from *R. nigrum L.* leaves contain numerous flavonoid glycosides, mainly derivatives of quercetin and kaempferol, and prodelphinidins^42^. Tabart et al. showed that blackcurrant extract has an antioxidant effect which is tolerated with the high content of phenolic compounds. What is more they showed the leaf was found to have a high flavonoid content mainly quercetin^17^. As is known, some flavonoids, like quercetin, rutin, resveratrol, are especially known for their antioxidant properties^43^. Leaves from *F. vesca L.* also contain numerous bioactive compounds, which include: phenolic acids (ellagic, p-coumaric, gallic acids), flavonoids (flavonols, quercetin, and kaempferol), proanthocyanidins, and anthocyanins (pelargonidin, cyanidin)^44–46^. Many studies have demonstrated the significant presence of various secondary metabolites in the leaves of *R. fruticosus L.,* such as: phenolic acids like ellagic, gallic, caffeic, and p-coumaric acids, flavonoids, such as quercetin, hyperoside, kaempferol, myricetin, catechin and epicatechin gallate^47–49^. What is more, Bujor et al. showed that the phenolic compounds contained in *Vaccinium myrtillus L.* are found in a much greater content in leaves and stems than in fruit. The most abundant amounts in the leaves were caffeic acid derivatives, their content ranged from 67 to 79% of the dry extract mass (DE), while flavanol oligomers stood out in their stems, constituting 54 to 62% of the total phenol content^50,51^. In addition, Roslon et al. have shown that the ethanol and water leaves extracts were significantly richer in polyphenols in comparison to the fruits extracts^52^. As demonstrated by Gawron-Gzella et al., antioxidant activity is positively correlated with the total content of phenolic compounds and phenolic acids^53^. Many authors have demonstrated that these raw plant materials exhibited high antioxidant activity and can be used in the food or pharmaceutical industry^38,54^.

The studies show that these extracts are a significant source of a natural antioxidant, which might be helpful in preventing the progress of various oxidative damages. Giacolone et al. have explained why blueberries are powerful intracellular antioxidants^55^. It is probable that polyphenols *V. myrtillus L.* beyond direct capture action strengthen endogenous antioxidants. It has been observed that during exposure, the amount of glutathione and ascorbic acid were increased in neurons, as well activity of antioxidants such as catalase and superoxide dismutase. Antioxidant activity of blueberry extracts was also associated with a reduction in ROS production and consequently, a reduction in their effects, including lipid peroxidation^56–58^. Jeong et al. have shown that an oxidative stress suppression by *V. myrtillus L.* polyphenols counteract the cytotoxic effect β-amyloid, which is an abnormal protein in the process of neurodegeneration in mice^59^.

### Cell viability assay

**Figure 2:**
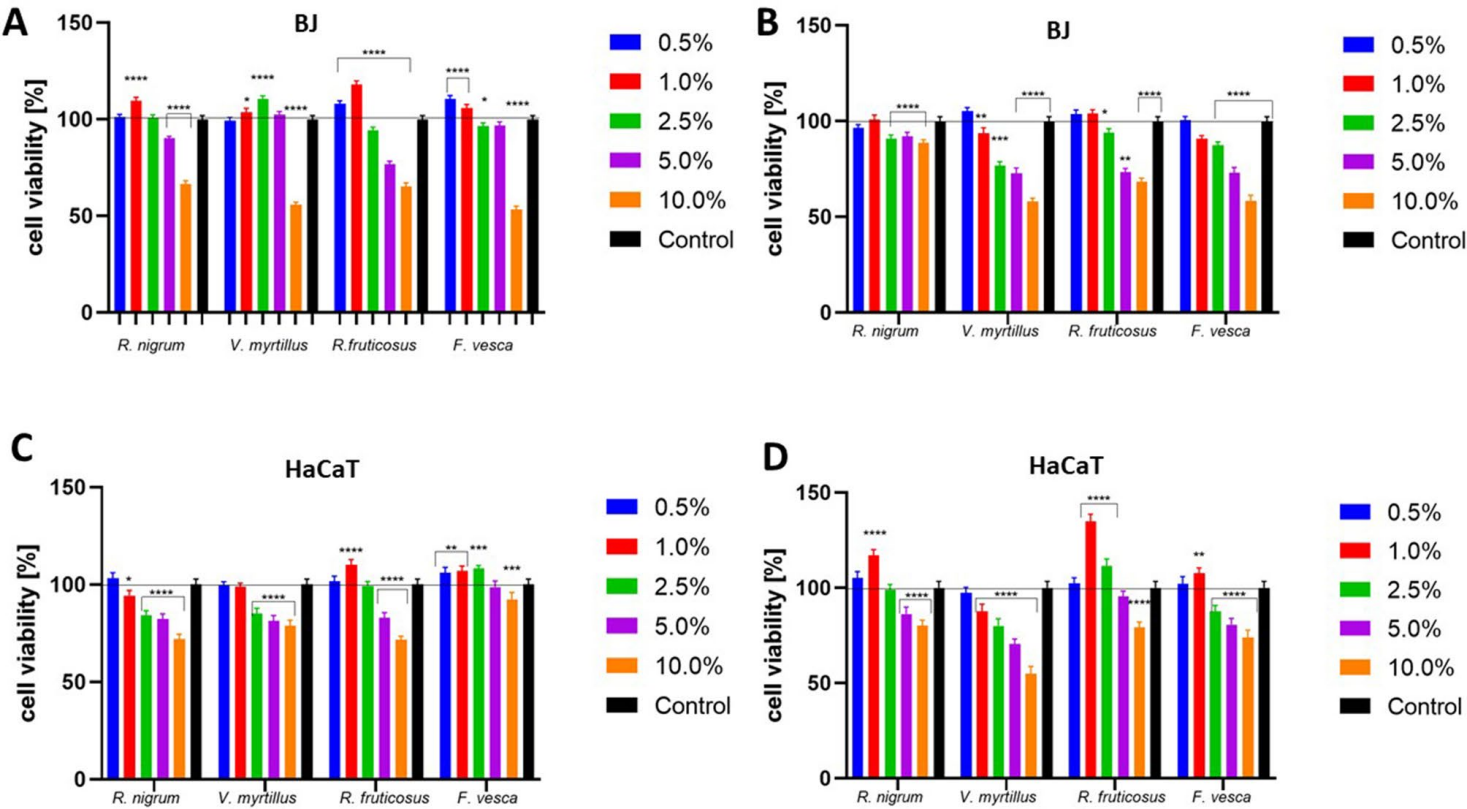
Cytotoxicity assessment of water-glycerin extracts from leaves of *Ribes nigrum L., Vaccinium myrtillus L, Rubus fruticosus L.* and *Fragaria vesca L*. on fibroblasts (BJ) (**A**,**B**) and keratinocytes (HaCaT) cells (**C**,**D**) after 24-h exposure assessed by the Alamar Blue assay (**A**,**C**) and Neutral Red assay (**B**,**D**). Data are the mean ± SD of three independent experiments in which each concentration tested was performed in four replications. The control was cells not treated with extracts, whose viability was assumed to be 100%. ****p < 0.0001, ***p < 0.001, **p < 0.01, *p < 0.03 versus the control (100%).

The unusual properties of leaf extracts of various plants, especially herbs or arable crops, have been known for a long time and have also been used in folk medicine^60^. In contrast, the leaves of the plants we study are very often considered as waste material, which is incomprehensible considering scientific studies assessing the amount of biologically active compounds in these leaves and the beneficial properties that these plants exhibit. Numerous studies indicate that their properties are even better than fruits, which are primarily used by consumers. The Alamar Blue assay used in this study is based on the change of the blue color of the non-fluorescent indicator dye (resazurin) after acceptance of electrons, which, passing from the oxidized state to the reduced state, becomes a fluorescent pink compound^61^. The analyzes carried out to assess the cytotoxic properties of the obtained extracts from the leaves of four tested plants have shown that these extracts have different effects on the tested skin cell lines. Due to the fact that keratinocytes are cells that are directly exposed to various external factors as well as cosmetic preparations applied directly to the skin, it is reasonable to perform cytotoxicity tests against these cells^62,63^. Especially if new cosmetic raw materials of natural origin, including the fruit plant leaves we examine, are analyzed for their potential use in cosmetic or pharmaceutical preparations. Our analyzes have shown that extracts from the leaves of *Ribes nigrum L., Vaccinium myrtillus L, Rubus fruticosus L.* and *Fragaria vesca L.* differ in cytotoxicity, both for keratinocytes and fibroblasts. The conducted analyzes clearly indicate that the effect exerted by the tested extracts is strictly dependent on the concentration used. Analyzes have shown that higher concentrations of leaf extracts inhibit the viability and metabolism of the cell lines tested. Cytotoxicity tests performed on keratinocytes (HaCaT) showed that the most beneficial effect on these cells is obtained by the leaves of *Rubus fruticosus L.* and *Fragaria vesca L*. The extract of *R. fruticosus L*. has a beneficial effect at a concentration of up to 1.0%, while in the case of *Fragaria vesca L*. the extract has a positive effect up to a concentration of 2.5%. Exposure of HaCaT cells to *R. nigrum L.* extracts shows beneficial effects only at a concentration of 0.5%, while *V. myrtillus L*. extract has no beneficial effect at any of the concentrations used. It is noticeable, however, that concentrations of all tested extracts equal to or greater than 5.0% reduce the proliferation and viability of keratinocytes, which indicates their cytotoxic effect associated with the reduction of cellular metabolism. The greatest inhibition of keratinocyte viability was observed for leaf extracts of *R. nigrum L.* and *R. fruticosus L*. at a concentration of 10.0%, where the inhibition reached almost 30% (Fig. 2). It should be noted here that the cytotoxic effect caused by the analyzed extracts in higher concentrations need not be associated with inhibition of only mitochondrial reductase. There are also other mitochondrial and cytoplasmic enzymes such as diaphoresis, NAD (P) H: quinone oxidoreductase, dihydrolipoamide dehydrogenase and flavin reductase, which are also able to reduce Alamar Blue. Therefore, the effect of inhibiting the viability of the cells studied is not necessarily associated with direct disruption of electron transport or causing mitochondrial dysfunction^64^.

The cytotoxic effect of the obtained extracts was also assessed using a neutral red assay. The principle of this test is based on the determination of viable cells by assessing the amount of dye taken into the lysosomes. These organelles in live cells can uptake this dye through active transport. In the case of the cytotoxic effect of the compounds tested, the cells are not able to incorporate this chromophore into the lysosomes^65^. The analyzes carried out confirmed the results obtained using the previous test. Analyzes aimed at determining the amount of neutral red incorporated in the lysosomal structures of HaCaT cells also indicated that these extracts at lower concentrations stimulate cell viability which was manifested by a greater amount of dye binded into lysosomes, while higher concentrations have a negative effect. The extracts from *R. nigrum L., R. fruticosus L.* and *F. vesca L.* at the lower concentrations used stimulate the viability of keratinocytes, whereas the extracts from *V. myrtillus L.* show no beneficial effect on these cells at any concentration. The most favorable concentration of studied extracts showing a positive effect seems to be a concentration of 1.0%, which in the case of the *R. fruticosus* L. extract can increase the viability of HaCaT cells by up to 35% (Fig. 2). A decrease in the amount of dye released from lysosomes treated with higher concentrations of extracts indicates a decrease in their viability, since during cell death the pH gradient decreases, which leads to the inability to retain the dye inside the lysosomes. Accordingly, it is assumed that the amount of dye retained is proportional to the number of viable cells. Moreover, changes in the amount of captured neutral red can also be the result of a change in the surface of cells and lysosomal membranes^66^.

As part of this work, the effect of the extracts obtained on other skin cells—fibroblasts—was also analyzed. These cells are located in the deeper layers of the skin compared to keratinocytes, however, due to the possibility of penetration of the ingredients of cosmetic and pharmaceutical preparations through the layers of the skin during their topical application, it is reasonable to examine the cytotoxic effect on these cells of natural compounds with potential application in these preparations. The analyzes carried out using the fibroblast cell line demonstrated similar results as in the case of keratinocytes. After performing the Alamar Blue test, a concentration-dependent effect was observed which differed depending on the plant being analyzed. Fibroblasts were found to be more sensitive to the action of the extracts obtained in the highest concentrations compared to keratinocytes. In *V. myrtillus L.* and *F. vesca L.,* 10.0% concentration resulted in almost 50% inhibition of cell viability. In contrast, lower concentrations of analyzed plants, mainly 0.5 and 1.0%, had a positive effect on the activity of mitochondrial and cytoplasmic enzymes involved in the reduction of resazurin (Fig. 2). In contrast, the Neutral Red test showed only a slight beneficial effect on fibroblasts caused by *V. myrtillus L.* and *R. fruticosus L.* at the lowest concentrations. Higher concentrations of all of the extracts used showed a negative effect on these cells, as indicated by less absorbance of neutral red after applying the decolorizing solution. A smaller amount of dye accumulated in lysosomes indicates a decrease in viability, because in intact cells this dye binds and accumulates in these organelles. This is because it becomes charged and cannot pass freely into the cytoplasm^67^. The cytotoxic effect of the higher concentrations of extracts observed during the experiments probably caused cell damage that resulted in the inability to accumulate dye inside the lysosomes. Hence, lower absorbance of the dye after its release with a decolorizing solution was observed (Fig. 2).

Analyzes evaluating the effect of extracts from various types of berries were carried out on both cell and normal lines. Although numerous studies in vitro*, *in vivo, and clinical studies indicate that fruit is a rich source of bioactive compounds, various authors try to prove that leaves can also be a valuable source of extremely valuable and therapeutic compounds. The research carried out as part of this work and the cited literature data show that extracts obtained from the leaves of these plants, which are mainly seen as waste material, show even better properties than extracts from fruits^64^. This was demonstrated by studies using gastric cancer cells (AGS line), where leaf extracts were more able to inhibit their proliferation. These studies also indicated that blueberry leaf extracts significantly limit the migration capacity of these cancer cells, which is caused to a large extent by the high content of anthocyanins in these extracts^64^. The significant antioxidant properties of these extracts play a significant role in this anti-cancer effect. Research conducted by Debnath-Canning et al. also pointed to the remarkable properties of blueberry extracts, demonstrating that they show a protective effect on microglia cells by protecting them against death and the ability to reduce the signs and morphological changes associated with neurological inflammation. These authors also indicated that leaf extracts are characterized by better activity than fruit extracts, which probably results from a greater number of biologically active compounds in the leaves, which was confirmed by HPLC–MS^68^. These studies indicate that the intake of blueberry leaves in the form of dried herbs, extracts, teas or as additives to medicinal products can have a neuroprotective function and play a large role in the chemoprevention of many different diseases, including inflammatory ones. Studies to assess the viability of two colon cell lines (HT29 and SW948) also showed the cytotoxicity of blackberry leaf extracts in a concentration-dependent manner. In these studies, the extract obtained from *R. caesius* leaves reduced the growth of cancer cells, affecting both their morphology and metabolism. LC–MS studies of the analyzed extracts additionally showed that the leaves of these plants contain a wide range of polyphenols, which due to the well-known antioxidant activity may play an important role here^69^. Research also indicates the extremely valuable properties and chemopreventive role of various berries in vivo^70^. A very important aspect that should be taken into account when using berry extracts is the dose, because although they contain many substances with valuable properties, their excess may have a negative impact on our health^71^. The results obtained as part of this work on skin cells in vitro also confirmed the dose-dependent cytotoxicity of leaf extracts of the studied plants. Numerous studies indicate that berries, being a rich source of bioactive compounds with antioxidant properties, can be helpful in preventing inflammatory diseases, cardiovascular diseases and play a significant role in chemoprevention^72^. Literature data also indicate that berry extracts can have a positive effect on fibroblasts, protecting them from oxidative damage. This action is closely related to the content of compounds present in these plants^73^. Other authors also confirm the positive effect of berries on keratinocyte damage caused by UV-B irradiation by reducing the level of reactive oxygen species, inflammatory cytokines and expression of matrix metalloproteinase-1 (MMP-1)^74^.

The different effects of individual plants on the proliferation of keratinocytes and fibroblasts observed in this work are probably caused by the different composition of biologically active compounds contained in individual extracts. Articles describing bioactive compounds in berry leaves indicate that they contain primarily various phenolic acids, flavonols, esters, anthocyanins and procyanidins. These parts of berry plants are also a rich source of chlorogenic acid^21^. This compound has a proven positive effect on the skin by increasing cell proliferation, affecting inflammatory processes, accelerating wound healing and collagen IV synthesis. It also has antioxidant properties, which is associated with an increase in superoxide dismutase, catalase and glutathione activity as well as a decrease in the degree of lipid peroxidation^75^. Literature data indicate that various compounds of plant origin have a different effect on cell proliferation, hence the content of individual compounds and interactions between them will strictly affect the final effect of the extracts^76^. Also, the concentration of individual compounds in the obtained extracts is of great importance for the effect of their action on cells, as numerous studies indicate a dose-dependent effect of compounds of plant origin^77^. The anthocyanins and anthocyanidins contained in the tested plants can also affect cell viability, because, as shown by studies by Wang et al., in low concentrations these compounds can cause arrest in the G0/G1 phase of the cell cycle, while at higher concentrations the cycle is inhibited in phase G2/M. Higher concentrations may also result in cell death—both necrotic and apoptotic. The induction of apoptosis by polyphenolic compounds is associated with the caspase family and the effect on p53, Bax, Bcl-2, Bcl-X1 levels^78^. Moreover, anthocyanins can also cause loss of mitochondrial membrane potential of apoptotic cells and typical PARP-1 degradation^79^. Literature data also indicate that compounds of plant origin may affect cell proliferation by acting as bioactive mediators and by participating in the regulation of cell division and differentiation. This effect is associated with the impact on complex signal paths such as, for example, BMP2, Runx2 and Wnt^77^. Thus, the different effect of individual extracts will probably be based on different proportions of individual groups of biologically active compounds in the extracts we have obtained. Due to the fact that there are only a few studies pointing to the fact that the leaves are equally valuable material that can show beneficial effects on various types of cells, including skin, this paper may increase interest in this plant material and expand its use, also in preparations intended for the care and treatment of skin diseases.

### Scratch wound assay

**Figure 3:**
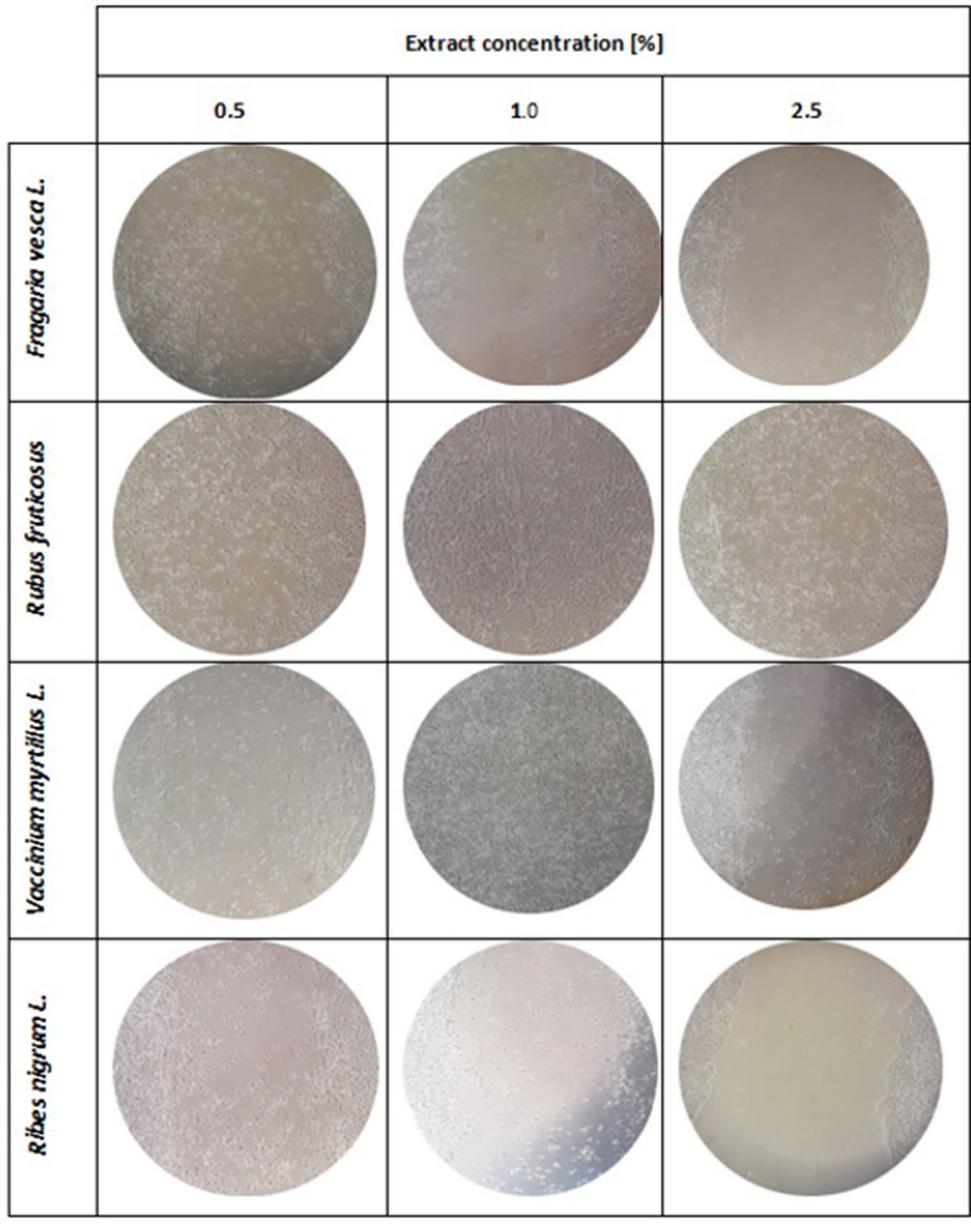
The effect of water-glycerin leaf extracts from *Ribes nigrum L., Vaccinium myrtillus L, Rubus fruticosus L.* and *Fragaria vesca L.* (at a concentration of 0.5–2.5%) on the migration of HaCaT cells after a 24-h incubation in a scratch test. Photographs were taken under a magnification of × 10 under a microscope. The analyzes were performed in triplicate from three independent experiments.

The next stage of the research was to assess the possibility of using the obtained leaf extracts as potential components of preparations that may find application in the treatment of wounds occurring in various skin diseases and resulting from the use of various methods of their treatment. The wound healing test used for this purpose is a test enabling evaluation of both migration and cell morphology in real time. This method is widely used in research on the process of wound healing, angiogenesis as well as the processes of metastasis of various types of cancer. This test is used by all to analyze the migration of cells, such as fibroblasts or keratinocytes, which are characterized by the ability to collective migration, also known as "sheet migration"^80^. The ability to stimulate migration by substances of natural origin is extremely desirable, because this property, in addition to well-known antioxidant properties, can have a positive effect on the condition and renewal of the skin after various dermatological and cosmetological treatments. The scratch test procedure involves performing a linear scratch in the monolayer of the tested cell line with appropriate confluence, and estimating cell migration resulting in gap clogging^81^. The assessment of the migration capacity of the cells, keratinocytes and fibroblasts tested was carried out after 24 h of incubation with the obtained water-glycerin extracts. This time was used to minimize the role of cell proliferation and be limited to assessing the ability of cells to migrate. As recommended, for this purpose, a lower FBS concentration (1%) in the culture medium (serum starvation) was used compared to the concentration used during normal keratinocyte and fibroblast culture (10%) (Vang Mouritzen and Jenssen, 2018). The results of the experiments carried out as part of this work showed that the analyzed extracts obtained from the leaves *of Ribes nigrum L., Vaccinium myrtillus L., Rubus fruticosus L.,* and *Fragaria vesca L.* affect the migration process of the tested cells to varying degrees. These results correlate with cytotoxicity tests, as it has been observed that the concentration of extracts that caused cytotoxic activity on keratinocytes or fibroblasts also inhibited the migration of these cells. The greatest ability to stimulate keratinocyte migration was demonstrated by the extract obtained from *R. fruticosus L.* leaves, which, when the concentration of 1.0% was used, resulted in almost complete closure of the gap. However, it was observed that higher concentration (5.0%) stimulates migration to a lesser extent and the number of cells in a break is smaller. Another plant showing the ability to induce HaCaT cell migration was *V. myrtillus L.*, which also showed the best activity at a concentration of 1%. For the other two extracts, no induction of migration was observed. Only at the lowest concentrations in the scratch made few cells were observed (Fig. 3). At higher concentrations (5.0% and 10.0%), morphological changes of cells and cell detachment from the surface of culture plates were observed, which confirms the above-demonstrated cytotoxic effect of higher extract concentrations (data not shown).

**Figure 4:**
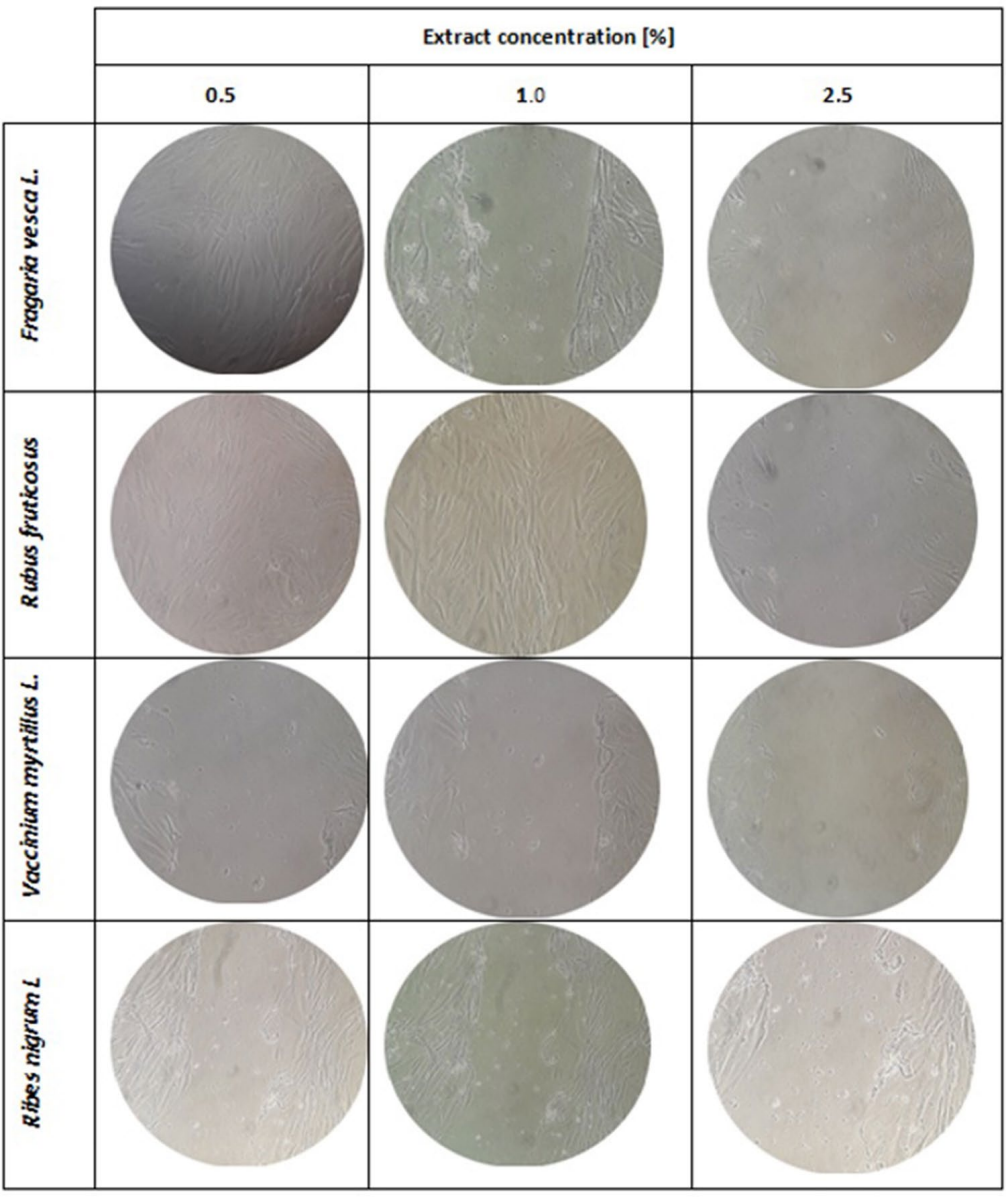
The effect of water-glycerin leaf extracts from *Ribes nigrum L., Vaccinium myrtillus L, Rubus fruticosus L.* and *Fragaria vesca L.* (at a concentration of 0.5–2.5%) on the migration of fibroblast cells after a 24-h incubation in a scratch test. Photographs were taken under a magnification of × 10 under a microscope. The analyzes were performed in triplicate from three independent experiments.

In the case of fibroblasts, it has also been observed that some of the extracts used may increase the proliferative capacity of these cells. This ability is characterized primarily by the extract of *R. fruticosus L.* leaves, which in both 0.5% and 1.0% concentration caused the migration of cells to the entire surface of the crack. Good results have also been observed after treatment of fibroblasts with 0.5% concentration of *F. vesca L*. In the case of *V. myrtillus L.* no increase in fibroblast migration was observed, whereas after using *R. nigrum L.* the increase was negligible (Fig. 4). As with keratinocytes, higher concentrations had a negative effect on cell morphology and adhesion (data not shown).

Although the antioxidant properties and content of biologically active compounds in the studied plants are well known, there are only a few studies on the possibility of these plants affecting cell migration processes and they focus primarily on fruit activity. Due to the large amount of biologically active compounds that occur in both the leaves and fruits of the plants studied, it seems reasonable to carry out research aimed at assessing the possibility of using leaf extracts in preparations used to accelerate the process of wound healing. Esposito et al. in their research on the effects of *Vaccinium uliginosum* extracts, they demonstrated that the compounds they contain promote wound closure and can affect early stages of healing by stimulating mitochondrial bioenergetics, as well as increasing the expression of important extracellular matrix proteins^82^. Our analyzes have shown that the best activity to stimulate the migration of cells, both fibroblasts and keratinocytes, has blackberry leaf extract. Other authors also pointed to the positive effect of these fruits on skin fibroblast migration processes, which, as they explain, was closely related to the high content of polyphenolic compounds, mainly anthocyanins^83^. The positive effect on the in vivo wound healing process caused by extracts from *R. nigrum* leaf has been shown by Kendir et al. Who associated this effect with well-known anti-microbial and anti-inflammatory properties, which plays an important role in wound healing^84^.

Cell migration is an important step in creating and repairing tissues. The proper migration of cells is an extremely important aspect necessary for the proper course of various processes in the human body. It is not only associated with wound repair, but also plays an important role in the immune response and maintaining tissue homeostasis. On the other hand, disorders in migration processes result in various pathological conditions, including tumor metastasis^83^. Thus, the scratch test is a technique used by many researchers conducting research on understanding the mechanisms of action of various compounds of medical importance on the process of wound healing in vitro^85^. Research is being conducted around the world to find the answer to what most impacts these processes. Undoubtedly, they depend on many factors, among which biochemical communication via paracrine signaling as well as gap connections are important^80^. The mobility and migration capacity of cells treated with various compounds or extracts also depend on their adhesive strength, mechanical flexibility as well as external migration signals and cues. Equal dimensions and organization of the cell cytoskeleton are important here^86–88^. Migration processes, which are a very important stage enabling the proper course of wound healing, are also closely dependent on the selective activation of cells at the edge of the wound and also by the cohesion forces occurring between the migrating cells^80,83^. The ways in which cells can move during the migration process can be different and depend on the type of cell being analyzed. Some of them can travel in groups consisting of cell chains and other forms of sheet-like layers^81^. However, it is worth noting here that some authors suggest that coherence and biochemical communication between migrating cells is not a prerequisite for observing this phenomenon. They suggest that the phenomenon of cell migration is associated with releasing these cells from mechanical constraints and minimizing the phenomenon of contact inhibition^80^.

Due to the few literature reports indicating the possibility of using extracts from the leaves of the studied plants in the wound healing process, the results obtained in this work may contribute to increasing interest in these waste materials. The high content of bioactive compounds (phenols and flavonoids), excellent antioxidant properties and a positive effect on the viability and migration of skin cells indicate that the obtained extracts are extremely valuable raw materials that can be widely used on the pharmaceutical and cosmetics market.

## Conclusion

The constantly growing expectations of consumers and their search for cosmetic products, which in addition to beneficial effects on their health will also have a positive impact on the environment, have contributed to an attempt to assess the possibility of using waste plant materials as a reservoir of extremely valuable bioactive compounds. The innovation in this work was mainly based on the choice as the raw material of the leaves of the analyzed berry plants, which, unfortunately, are underestimated in various industries, including the cosmetics industry. This choice also allowed to minimize the use of natural resources, since those plant parts that were usually seen as waste material were used to produce these extracts. Of course, the analyzed raw materials were widely used in traditional medicine, but they are still niche products in the cosmetics industry. The research additionally showed that the extracts are multifunctional, have a wide spectrum of health-promoting properties, and are also safe to use, which significantly expands their range of applications. Our results revealed that all investigated extracts from leaves of *R. nigrum L., V. myrtillus L., R. fruticosus L., F. vesca L.* were rich in phenolic compounds and demonstrated good antioxidant activity. The substances contained in the leaves of analyzed plants, particularly polyphenolic compounds, have beneficial properties for health. These substances could have potential use in the prevention of lifestyle diseases. The results have shown that lower concentrations of analyzed plants, mainly 0.5 and 1.0%, had a positive effect on fibroblasts and keratinocytes. Higher concentrations of all extracts used have shown a negative effect on these skin cells. In summary, these plants can be a valuable source of natural antioxidants and are a promising source of bioactive substances that can be used in the cosmetics industry as an ingredient in anti-aging cosmetics.
